# Case Report: Cerebral Venous Sinus Thrombosis and COVID-19 Infection

**DOI:** 10.3389/fmed.2021.741594

**Published:** 2021-10-15

**Authors:** Manasa Anipindi, Amanda Scott, Li Joyce, Salman Wali, Mark Morginstin

**Affiliations:** Einstein Healthcare Network, Philadelphia, PA, United States

**Keywords:** case report, cerebral venous sinus thrombosis (CVST), COVID-19, headache, anticoagulant

## Abstract

Coronavirus disease-2019 is caused by the severe acute respiratory syndrome coronavirus 2 (SARS-CoV-2 virus). Coronavirus disease-2019 (COVID-19) was declared a pandemic in March 2020 and has changed our lives in many ways. This infection induces a hypercoagulable state leading to arterial and venous thrombosis, but the exact pathophysiology of thrombosis is unknown. However, various theories have been postulated including excessive cytokine release, endothelial activation, and disseminated intravascular coagulation (DIC). We present a patient diagnosed with cerebral venous sinus thrombosis (CVST) with COVID-19 infection. A 66-year-old man presented to a hospital for evaluation of persistent headaches. He tested positive for COVID-19, and MRI of the brain and CT venogram revealed CVST. He was started on heparin drip in the hospital and transitioned to oral anticoagulants at the time of discharge. His headaches improved with treatment. Even though headache is the most frequent and initial symptom of cerebral venous thrombosis, it is rarely the only symptom. A high index of suspicion is therefore required to diagnose CVST especially if the patient presents with a simple complaint like a headache. Common complaints can delay the diagnosis leading to disease progression. Considering the high mortality rates in patients diagnosed with CVST, we suggest the importance of knowing the association between COVID-19 infection and CVST, especially in susceptible patients.

## Introduction

The coronavirus disease-2019 (COVID-19) pandemic has affected nearly every individual across the world. Thus far, the severe acute respiratory syndrome coronavirus 2 (SARS-CoV2) virus infection has infected approximately 180 million individuals and has resulted in 4 million deaths. The transmission of the virus is through direct contact and by airborne droplets (1). The virus attaches to a site on the ACE2 receptor and then replicates in the cytoplasm of the cell, producing progeny virions (2, 3). These virions are released from the cell into surrounding tissues of the respiratory tract (4). They are usually shed by coughing or sneezing and the incubation period of the infection is between 4.5 and 5.8 days (5). ACE2 receptors are present in many organs and tissues including the lungs, trachea, bronchi, stomach, small intestine, sweat glands, parathyroid, pituitary, pancreas, and cerebellar endothelial cells (6, 7).

Clinical manifestations of COVID-19 range from an asymptomatic state to multiorgan dysfunction (8). COVID-19 affects the respiratory system causing progressive respiratory failure and organ dysfunction, leading to generalized coagulopathy (9–11). Most commonly, infection manifests with symptoms including cough, fever, diarrhea, fatigue, headaches, and myalgias. Shortness of breath due to pneumonia is the most common symptom of hospital admission (12, 13). Respiratory failure, acute respiratory distress syndrome (ARDS), cardiac arrhythmias, coagulopathy, and shock are delayed manifestations of severe disease (14). Severe disease and progression are secondary to the release of inflammatory cytokines including interleukins (IL-2, IL-6, IL 7, IL 10), Granulocyte Colony Stimulating Factor (G-CSF), Interferon gamma-induced protein 10 (IP-10), MCD 1, M1 P18, and TNF alpha (15, 16).

Autopsies of infected patients have shown that the virus can cause microvascular injury in the brain (17). The mechanism by which the virus spreads to the brain is via the cribriform plate into areas surrounding the olfactory bulb, olfactory nerve, and through the blood-brain barrier (18–20). Neurological manifestations of Covid-19 range from headache to encephalopathy. The symptoms include headaches, dizziness, ageusia, weakness, and confusion. Patients with central nervous system involvement can be diagnosed with acute encephalopathy, acute cerebrovascular problems, acute ischemic stroke, hypoxia, Guillain-Barré syndrome (GBS), ataxia, olfactory disorders, gustatory dysfunction, seizures, psychosis, and cerebral venous sinus thrombosis (CVST) (21, 22).

## Cerebral Venous Sinus Thrombosis

Cerebral venous sinuses are an uncommon location of venous thrombosis and are mostly observed in women and the younger population (23–25). Common causes of CVST include hereditary prothrombotic conditions, antiphospholipid antibody syndrome (APLS), cancer, pregnancy, autoimmune diseases, and infections (26). It can lead to increased intracranial pressure, encephalopathy, strokes, cranial nerve palsies, seizures, and headaches (27). The most common presentation of CVST is a headache and it can be the only symptom in some instances (26, 28–31). The etiology of headache is likely secondary to the compression of nerves within veins (32), increased intracranial pressure, or, sometimes, venous infarct (33, 34). It is often difficult to differentiate CVST-induced headaches from other primary headache disorders. Most headaches caused by CVST are subacute in onset, causing diffuse throbbing pain. Other associated neurological findings include neurologic deficits, photophobia, or signs of increased intracranial pressure (35–38). Sometimes CVST can also lead to damage of brain parenchyma (39).

Diagnosing CVST can be very difficult due to differences in clinical presentation and imaging findings. Only about 25% of patients with CVST can be diagnosed by unenhanced head CT. CT venography and MRI can be used if there is a high suspicion after unenhanced head CT. The European Academy of Neurology recommends intravenous heparin or subcutaneous low molecular weight heparin first followed by oral anticoagulation for at least 3 to 12 months depending on the etiology of CVST (31).

## Cvst and COVID-19

Coronavirus disease-2019 infection causes a hypercoagulable state, resulting in increased inflammatory markers like D-dimer, Lactate dehydrogenase (LDH), ferritin, and C-reactive protein (CRP). It also causes an increase in clotting times (40–44). Usually, prothrombotic events including deep vein thrombosis (DVT) and pulmonary embolism occur later as the disease progresses. Many studies have shown an association of COVID-19 infection with CVST (45–48). The most common location of CVST secondary to COVID-19 infection is the transverse sinus followed by the sigmoid sinus (49). The severe microvascular injury in COVID-19 infection is suggested to be due to the combined effect of complement activation and hypercoagulability, leading to microvascular thrombosis. The cerebrovascular effects also seem to be secondary to hypercoagulability and endothelial injury resulting from the release of pro-inflammatory cytokines. Some patients diagnosed with CVST in COVID-19 infection present elevated anticardiolipin IgM antibody and lupus anticoagulant. They also had increased fibrinogen levels, inflammatory markers, and prothrombin time (50–57).

## Case Discussion

A 66-year-old man with a past medical history significant for GERD, hypothyroidism, previous head and neck cancer status, post-resection history chemotherapy, and radiation presented to the hospital for evaluation of headaches. The patient reported waking from sleep with a pounding headache two to three times per month over a 6-month period. The headaches were associated with palpitations, dizziness, and diaphoresis. He denied any complaints of blurred vision, slurred speech, weakness, numbness, or tingling of his extremities, nausea, and vomiting. He reported no complaints of shortness of breath, cough, nasal congestion, fatigue, or diarrhea. His pain was reportedly resolved by two to three tablets of ibuprofen 200 mg. While his headaches did not significantly impact his activities of daily living, they did prompt discussion with his primary care physician. An outpatient MRI brain was performed, which revealed findings suspicious of CVST. Thus, he was sent to the emergency department for further evaluation.

**Figure 1:**
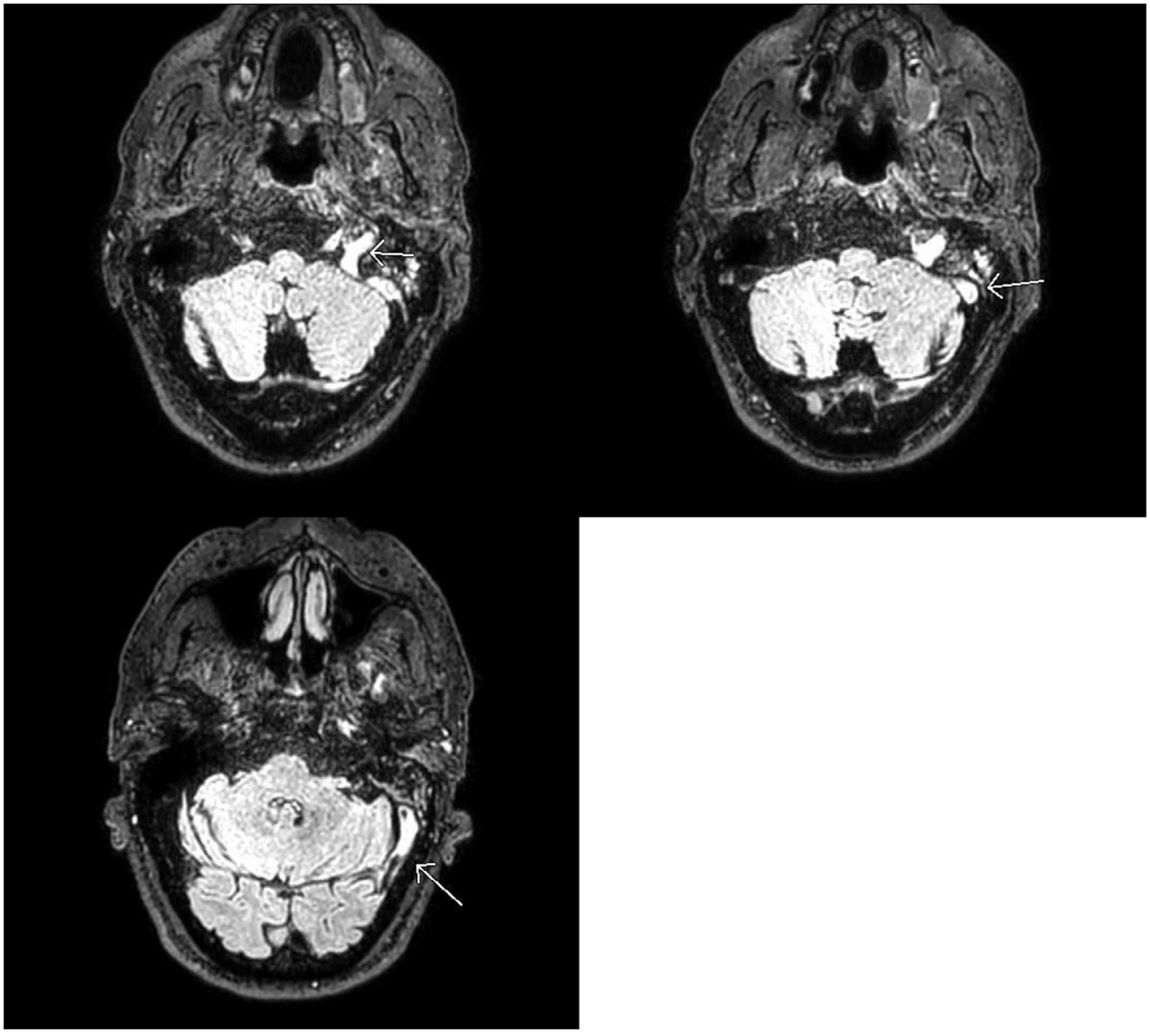
MRI Brain: Abnormal FLAIR signal in the distal left transverse and sigmoid sinuses as well as the jugular bulb with loss of normal flow void. No corresponding signal abnormality on T1 or gradient weighted sequences (not shown). Findings favored to be slow flow but can be seen with dural venous sinus thrombosis in the setting of headaches. If there is clinical concern for dural venous sinus thrombosis, CT, or MR venogram should be considered for definitive diagnosis.

**Figure 2:**
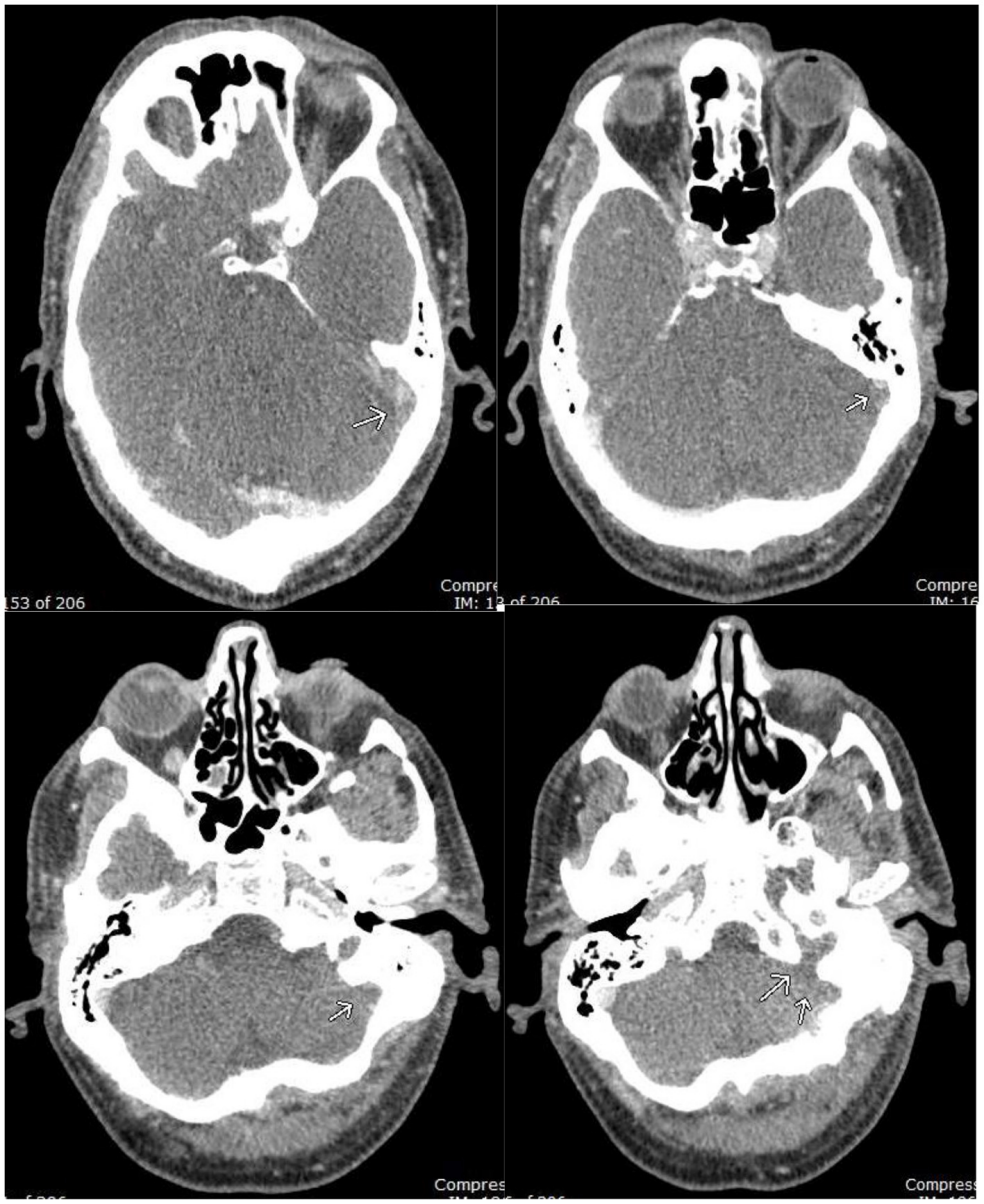
CT Venogram: filling defect in the left distal transverse sinus, sigmoid sinus, and jugular bulb, compatible with occlusive dural venous sinus thrombosis.

Three months prior to admission, the patient was tested for SARS-CoV-2 virus infection from a potential exposure at work. He tested positive and exhibited symptoms of cough, headache, and nasal congestion. He never exhibited significant shortness of breath or pleuritic chest pain. His symptoms improved with basic medical management. On his current admission, he was again tested for COVID-19 and tested positive. He denied a history of smoking, alcohol abuse, or recreational drug use. He had a past medical history of cancer on the base of the left tongue involving the left tonsils and left neck lymph nodes which was treated by surgery, radiation, and chemotherapy 11 years prior. He never presented any signs or symptoms of recurrence. Family history was not significant. His body mass index at the time of admission was 28 kg/m^2^, vital signs were within normal limits, and he was saturating well on room air. Physical examination including complete neurological examination was within normal limits. CBC revealed hemoglobin of 12.3, BMP revealed mild elevation of potassium was 5.2 mg/dl, TSH was within normal limits. His IgG, IgM, and IgA beta-2 glycoprotein antibodies were all within normal limits. IgG, IgA cardiolipin antibodies were within normal limits, but IgM was mildly positive at 21. Factor V Leiden and prothrombin gene mutations were negative. On telemetry, the patient had a pattern of bigeminy and trigeminy. He had no complaints of chest discomfort; there were no signs of ischemia, and a subsequent two-dimensional echo was within normal limits. CT brain imaging without contrast revealed no acute intracranial hemorrhage, midline shift, or mass effect but showed parenchymal volume loss, which was a probable sequelae of chronic small vessel ischemic change, as expected for age of the patient. There was no evidence of recurrence of his known cancer. MRI brain findings showed an abnormal FLAIR signal with loss of flow void in the distal left transverse sinus, sigmoid sinus, and jugular bulb (Figure 1). This was followed by a CT venogram, which revealed a filling defect in the left sigmoid sinus, jugular bulb, and visualized left internal jugular vein compatible with dural venous sinus thrombosis (Figure 2). He was started on intravenous heparin for dural sinus venous thrombosis. Prior to discharge, he has transitioned to Rivaroxaban 20 mg with anticipated treatment lasting 6 months. His symptoms had resolved at the follow-up appointment 3 months later.

## Discussion

Coronavirus disease-2019 usually manifests as fever, dyspnea, cough, diarrhea, and fatigue. The most common thromboembolic complications with COVID-19 infection are DVT and pulmonary emboli (47, 58). CVST is a rare thromboembolic event in the setting of COVID-19 disease, but the neurologic complications can sometimes be very severe if not diagnosed and treated early in the disease course (59, 60). The amount of CVST burden in hospitalized patients with SARS-CoV-2 infection was 0.08% with a 95% CI of 0.01 to −0.5 in a meta-analysis. It was also noted in the same study that risk factors other than SARS-CoV-2 infection were present in 31% of these individuals (59). Most patients presenting with CVST do not have a history of thrombotic disorders (58). Hereditary and acquired prothrombotic conditions like factor V Leiden, G20210A prothrombin gene mutation, malignancy, oral contraceptive use, pregnancy, and infection increase the risk of CVST compared to the general population (23). CVST complication in COVID-19 positive patients can present as loss of consciousness (37, 61), upward gaze (37, 61), visual deficits (60), convulsions (62), hemiparesis (62), headache (62, 63), executive dysfunction (63), dyspraxia (63), tongue biting (61), altered mentation (64), and aphasia (64). However, headache is the most common presenting symptom in patients with CVST (48). With advances in neuroimaging modalities, diagnosing CVST has become straightforward, however, early recognition of CVST is often obscured due to a wide variety of symptoms. Our case report presents headache as a manifestation of CVST associated with COVID-19. CVST can have symptoms from very mild to very severe. Headache is a common symptom in most viral illnesses and headache syndromes, therefore, a thorough evaluation is needed to rule out common problems before proceeding with expensive imaging studies. But new-onset recurring headaches need a high index of suspicion for treatable conditions like CVST as we can improve outcomes in such patients. Our patient had a previous history of cancer, and about 7–10% of patients diagnosed with CVST have cancer (65). Even though it is unlikely that his treated cancer which is in remission caused CVST, it is important to note that cancer increases the risk of thrombosis (66). The mortality rate of CVST associated with COVID-19 virus infection is significantly high (67, 68). Thus, in this pandemic era, even a symptom as common as a new-onset persistent headache should be investigated further as it may sometimes represent a medical emergency. In conclusion, timely recognition and awareness of this association enable prompt diagnosis and treatment with good clinical outcomes.

## Data Availability Statement

The original contributions presented in the study are included in the article/supplementary material, further inquiries can be directed to the corresponding authors.

## Ethics Statement

Written informed consent was obtained from the individual(s) for the publication of any potentially identifiable images or data included in this article.

## Author Contributions

MA wrote the case report under the guidance of MM. MM is the Hematologist who treated the patient. SW is the Neurologist involved in the care of the patient. LJ and AS from radiology were responsible for the interpretation of images. All authors were involved in making appropriate changes as needed and approved the final case report.

## Conflict of Interest

The authors declare that the research was conducted in the absence of any commercial or financial relationships that could be construed as a potential conflict of interest.

## Publisher's Note

All claims expressed in this article are solely those of the authors and do not necessarily represent those of their affiliated organizations, or those of the publisher, the editors and the reviewers. Any product that may be evaluated in this article, or claim that may be made by its manufacturer, is not guaranteed or endorsed by the publisher.

## Abbreviations

CVST: Cerebral venous sinus thrombosis
GERD: Gastroesophageal reflux disease
DVT: Deep vein thrombosis
APLS: Antiphospholipid syndrome
BMI: Basal Metabolic Index
CBC: Complete Metabolic Panel
BMP: Basal Metabolic Panel
LDH: Lactate dehydrogenase
CRP: C-Reactive protein
MRI: Magnetic resonance imaging
CT: Computed tomography.

## References

[1] Subbarao K Mahanty S. Respiratory virus infections: understanding COVID-19. Immunity. (2020) 52:905–9. 10.1016/j.immuni.2020.05.004 32497522 PMC7237932

[2] Cuervo NZ Grandvaux N. ACE2: Evidence of role as entry receptor for SARS-CoV-2 and implications in comorbidities. Elife. (2020) 9:e61390. 10.7554/eLife.61390 33164751 PMC7652413

[3] Shang J Ye G Shi K Wan Y Luo C Aihara H. Structural basis of receptor recognition by SARS-CoV-2. Nature. (2020) 581:221–4. 10.1038/s41586-020-2179-y 32225175 PMC7328981

[4] Fehr AR Perlman S. Coronaviruses: an overview of their replication and pathogenesis. Coronaviruses. (2015) 1–23. 10.1007/978-1-4939-2438-7_1 25720466 PMC4369385

[5] Lauer SA Grantz KH Bi Q Jones FK Zheng Q Meredith HR Azman AS. The incubation period of coronavirus disease (2019). (COVID-19) from publicly reported confirmed cases: estimation and application. Annals of Internal Medicine. (2020) 172:577–82. 10.7326/M20-0504 32150748 PMC7081172

[6] Ding Y He LI Zhang Q Huang Z Che X Hou J. Organ distribution of severe acute respiratory syndrome (SARS) associated coronavirus (SARS-CoV) in SARS patients: implications for pathogenesis and virus transmission pathways. J Pathol. (2004) 203:622–30. 10.1002/path.1560 15141376 PMC7167761

[7] Hamming I Timens W Bulthuis ML Lely AT Navis GV van Goor H. Tissue distribution of ACE2 protein, the functional receptor for SARS coronavirus. A first step in understanding SARS pathogenesis. J Pathol. (2004) 203:631–7. 10.1002/path.1570 15141377 PMC7167720

[8] Centers for Disease Control and Prevention (2021). Available online at: https://stacks.cdc.gov/view/cdc/89980

[9] Li X. Ma X. Acute respiratory failure in COVID-19: is it “typical” ARDS?. Critical Care. (2020) 24:1–5. 10.1186/s13054-020-02911-9 32375845 PMC7202792

[10] Zaim S Chong JH Sankaranarayanan V Harky A. COVID-19 and multi-organ response. Curr Probl Cardiol. (2020) 28:100618. 10.1016/j.cpcardiol.2020.100618 32439197 PMC7187881

[11] Al-Samkari H Karp Leaf RS Dzik WH Carlson JC Fogerty AE Waheed A. COVID-19 and coagulation: bleeding and thrombotic manifestations of SARS-CoV-2 infection. Blood. (2020) 136:489–500. 10.1182/blood.2020006520 32492712 PMC7378457

[12] Zhou F Yu T Du R Fan G Liu Y Liu Z. Clinical course and risk factors for mortality of adult inpatients with COVID-19 in Wuhan, China: a retrospective cohort study. Lancet. (2020) 395:1054–62. 10.1016/S0140-6736(20)30566-3 32171076 PMC7270627

[13] Wang D Hu B Hu C Zhu F Liu X Zhang J. Clinical characteristics of 138 hospitalized patients with 2019 novel coronavirus–infected pneumonia in Wuhan, China. Jama. (2020) 323:1061–9. 10.1001/jama.2020.1585 32031570 PMC7042881

[14] Guo T Fan Y Chen M Wu X Zhang L He T. Cardiovascular implications of fatal outcomes of patients with coronavirus disease 2019 (COVID-19). JAMA cardiology. (2020) 5:811–8. 10.1001/jamacardio.2020.1017 32219356 PMC7101506

[15] Singhal T. A review of coronavirus disease-2019 (COVID-19). Indian J Pediatr. (2020) 87:281–6. 10.1007/s12098-020-03263-6 32166607 PMC7090728

[16] Moore JB June CH. Cytokine release syndrome in severe COVID-19. Science. (2020) 368:473–4. 10.1126/science.abb8925 32303591

[17] Lee MH Perl DP Nair G Li W Maric D Murray H. Microvascular injury in the brains of patients with Covid-19. N Engl J Med. (2021) 384:481–3. 10.1056/NEJMc2033369 33378608 PMC7787217

[18] Cheng Q Yang Y Gao J. Infectivity of human coronavirus in the brain. EBioMedicine. (2020) 56:102799. 10.1016/j.ebiom.2020.102799 32474399 PMC7255711

[19] Baig AM Khaleeq A Ali U Syeda H. Evidence of the COVID-19 virus targeting the CNS: tissue distribution, host–virus interaction, and proposed neurotropic mechanisms. ACS Chem Neurosci. (2020) 11:995–8. 10.1021/acschemneuro.0c00122 32167747

[20] Meinhardt J Radke J Dittmayer C Franz J Thomas C Mothes R. Olfactory transmucosal SARS-CoV-2 invasion as a port of central nervous system entry in individuals with COVID-19. Nat Neurosci. (2021) 24:168–75. 10.1038/s41593-020-00758-5 33257876

[21] Zubair AS McAlpine LS Gardin T Farhadian S Kuruvilla DE Spudich S. Neuropathogenesis and neurologic manifestations of the coronaviruses in the age of coronavirus disease 2019: a review. JAMA Neurol. (2020) 77:1018–27. 10.1001/jamaneurol.2020.2065 32469387 PMC7484225

[22] Wildwing T Holt N. The neurological symptoms of COVID-19: a systematic overview of systematic reviews, comparison with other neurological conditions and implications for healthcare services. Ther Adv Chronic Dis. (2021) 12:2040622320976979. 10.1177/2040622320976979 33796241 PMC7970685

[23] Ageno W Beyer-Westendorf J Garcia DA Lazo-Langner A McBane RD Paciaroni M. Guidance for the management of venous thrombosis in unusual sites. J Thromb Thrombolysis. (2016) 41:129–43. 10.1007/s11239-015-1308-1 26780742 PMC4715841

[24] Coutinho JM Zuurbier SM Aramideh M Stam J. The incidence of cerebral venous thrombosis: a cross-sectional study. Stroke. (2012) 43:3375–7. 10.1161/STROKEAHA.112.671453 22996960

[25] Silvis SM Lindgren E Hiltunen S Devasagayam S Scheres LJ Jood K. Postpartum period is a risk factor for cerebral venous thrombosis: a case-control study. Stroke. (2019) 50:501–3. 10.1161/STROKEAHA.118.023017 30621526

[26] Green M Styles T Russell T Sada C Jallow E Stewart J. Non-genetic and genetic risk factors for adult cerebral venous thrombosis. Thromb Res. (2018) 169:15–22. 10.1016/j.thromres.2018.07.005 30005273

[27] Ferro JM Canhão P Stam J Bousser MG Barinagarrementeria F. Prognosis of cerebral vein and dural sinus thrombosis: results of the International Study on Cerebral Vein and Dural Sinus Thrombosis (ISCVT). Stroke. (2004) 35:664–70. 10.1161/01.STR.0000117571.76197.26 14976332

[28] Zuurbier SM Middeldorp S Stam J Coutinho JM. Sex differences in cerebral venous thrombosis: a systematic analysis of a shift over time. Int J Stroke. (2016) 11:164–70. 10.1177/1747493015620708 26783307

[29] Zuurbier SM Hiltunen S Lindgren E Silvis SM Jood K. Cerebral venous thrombosis in older patients. Stroke. (2018) 49:197–200. 10.1161/STROKEAHA.117.019483 29203685

[30] Ameri A Bousser MG. Cerebral venous thrombosis. Neurol Clin. (1992) 10:87–111. 10.1016/S0733-8619(18)30235-4 1557011

[31] Ferro JM Bousser MG Canhão P Coutinho JM Crassard I Dentali F. European Stroke Organization guideline for the diagnosis and treatment of cerebral venous thrombosis–endorsed by the European Academy of Neurology. Eur Stroke J. (2017) 2:195–221. 10.1177/2396987317719364 31008314 PMC6454824

[32] Mahale R Mehta A John AA Buddaraju K Shankar AK Javali M.. Acute seizures in cerebral venous sinus thrombosis: what predicts it? Epilepsy Res. (2016) 123:1–5. 10.1016/j.eplepsyres.2016.01.011 27023399

[33] Kalita J Misra UK Singh VK Dubey D. Predictors and outcome of status epilepticus in cerebral venous thrombosis. J Neurol. (2019) 266:417–25. 10.1007/s00415-018-9145-8 30569383

[34] Kowoll CM Kaminski J Weiß V Bösel J Dietrich W Jüttler E. Severe cerebral venous and sinus thrombosis: clinical course, imaging correlates, and prognosis. Neurocrit Care. (2016) 25:392–9. 10.1007/s12028-016-0256-8 27000641

[35] Lee KR Subrayan V Win MM Mohamad NF Patel D. ATRA-induced cerebral sinus thrombosis. J Thromb Thrombolysis. (2014) 38:87–9. 10.1007/s11239-013-0988-7 24046068

[36] Coutinho JM Zuurbier SM Gaartman AE Dikstaal AA Stam J Middeldorp S. Association between anemia and cerebral venous thrombosis: case–control study. Stroke. (2015) 46:2735–40. 10.1161/STROKEAHA.115.009843 26272383

[37] Buyck PJ De Keyzer F Vanneste D Wilms G Thijs V Demaerel P. density measurement and H: H ratio are useful in diagnosing acute cerebral venous sinus thrombosis. Am J Neuroradiol. (2013) 34:1568–72. 10.3174/ajnr.A3469 23471024 PMC8051442

[38] Rodallec MH Krainik A Feydy A Hélias A Colombani JM Jullès MC. Cerebral venous thrombosis and multidetector CT angiography: tips and tricks. Radiographics. (2006) 26:S5–18. 10.1148/rg.26si065505 17050519

[39] Fu FW Rao J Zheng YY Song L Chen W Zhou QH. Perimesencephalic nonaneurysmal subarachnoid hemorrhage caused by transverse sinus thrombosis: a case report and review of literature. Medicine. (2017) 96:e7374. 10.1097/MD.0000000000007374 28816935 PMC5571672

[40] Lippi G Favaloro EJ. D-dimer is associated with severity of coronavirus disease 2019: a pooled analysis. Thromb Haemost. (2020) 120:876. 10.1055/s-0040-1709650 32246450 PMC7295300

[41] Xie Y Wang X Yang P Zhang S. COVID-19 complicated by acute pulmonary embolism. Radiology: Cardiothoracic Imaging. (2020) 2:e200067. 10.1148/ryct.2020200067 33778561 PMC7233431

[42] Yin S Huang M Li D Tang N. Difference of coagulation features between severe pneumonia induced by SARS-CoV2 and non-SARS-CoV2. J Thromb Thrombolysis. (2021) 51:1107–10. 10.1007/s11239-020-02105-8 32246317 PMC7124128

[43] Guan WJ Ni ZY Hu Y Liang WH Ou CQ He JX. Clinical characteristics of coronavirus disease 2019 in China. N Engl J Med. (2020) 382:1708–20. 10.1056/NEJMoa2002032 32109013 PMC7092819

[44] Kuba K Imai Y Rao S Gao H Guo F Guan B. A crucial role of angiotensin converting enzyme 2 (ACE2) in SARS coronavirus–induced lung injury. Nat Med. (2005) 11:875–9. 10.1038/nm1267 16007097 PMC7095783

[45] Cavalcanti DD Raz E Shapiro M Dehkharghani S Yaghi S Lillemoe K. Cerebral venous thrombosis associated with COVID-19. Am J Neuroradiol. (2020) 41:1370–6. 10.3174/ajnr.A6644 32554424 PMC7658892

[46] Abdalkader M Shaikh SP Siegler JE Cervantes-Arslanian AM Tiu C Radu RA. Cerebral Venous Sinus Thrombosis in COVID-19 Patients: A Multicenter Study and Review of Literature. J Stroke Cerebrovascular Dis. (2021) 4:105733. 10.1016/j.jstrokecerebrovasdis.2021.105733 33743411 PMC7931726

[47] Al-Mufti F Amuluru K Sahni R Bekelis K Karimi R Ogulnick J. Cerebral venous thrombosis in COVID-19: A New York Metropolitan Cohort Study. Am J Neuroradiol. (2021) 42:1196–200. 10.3174/ajnr.A7134 33888450 PMC8324265

[48] Dakay K Cooper J Bloomfield J Overby P Mayer SA Nuoman R. Cerebral venous sinus thrombosis in COVID-19 infection: a case series and review of the literature. J Stroke Cerebrovascular Dis. (2020) 6:105434. 10.1016/j.jstrokecerebrovasdis.2020.105434 33190109 PMC7833244

[49] Tu TM Goh C Tan YK Leow AS Pang YZ Chien J. Cerebral venous thrombosis in patients with COVID-19 infection: a case series and systematic review. J Stroke Cerebrovascular Dis. (2020) 6:105379. 10.1016/j.jstrokecerebrovasdis.2020.105379 33254369 PMC7538072

[50] Teuwen LA Geldhof V Pasut A Carmeliet P. COVID-19: the vasculature unleashed. Nature Rev Immunol. (2020) 20:389–91. 10.1038/s41577-020-0343-0 32439870 PMC7240244

[51] Libby P Lüscher T. COVID-19 is, in the end, an endothelial disease. Eur Heart J. (2020) 41:3038–44. 10.1093/eurheartj/ehaa623 32882706 PMC7470753

[52] Middleton EA He XY Denorme F Campbell RA Ng D Salvatore SP. Neutrophil extracellular traps contribute to immunothrombosis in COVID-19 acute respiratory distress syndrome. Blood. (2020) 136:1169–79. 10.1182/blood.2020007008 32597954 PMC7472714

[53] Panigada M Bottino N Tagliabue P Grasselli G Novembrino C Chantarangkul V. Hypercoagulability of COVID-19 patients in intensive care unit: a report of thromboelastography findings and other parameters of hemostasis. J Thrombosis Haemostasis. (2020) 18:1738–42. 10.1111/jth.14850 32302438 PMC9906150

[54] Ranucci M Ballotta A Di Dedda U Bayshnikova E Dei Poli M Resta M. The procoagulant pattern of patients with COVID-19 acute respiratory distress syndrome. J Thrombosis Haemostasis. (2020) 18:1747–51. 10.1111/jth.14854 32302448 PMC9906332

[55] Maier CL Truong AD Auld SC Polly DM Tanksley CL Duncan A. COVID-19-associated hyperviscosity: a link between inflammation and thrombophilia? Lancet. (2020) 395:1758–9. 10.1016/S0140-6736(20)31209-5 32464112 PMC7247793

[56] Medicherla CB Pauley RA de Havenon A Yaghi S Ishida K Torres JL. Cerebral venous sinus thrombosis in the COVID-19 pandemic. J Neuro-Ophthalmol. (2020) 40:457–62. 10.1097/WNO.0000000000001122 33186264

[57] Gavriilaki E Anyfanti P Gavriilaki M Lazaridis A Douma S Gkaliagkousi E. Endothelial dysfunction in COVID-19: lessons learned from coronaviruses. Curr Hypertens Rep. (2020) 22:1–2. 10.1007/s11906-020-01078-6 32852642 PMC7449866

[58] Lodigiani C Iapichino G Carenzo L Cecconi M Ferrazzi P Sebastian T. Venous and arterial thromboembolic complications in COVID-19 patients admitted to an academic hospital in Milan, Italy. Thromb Res. (2020) 191:9–14. 10.1016/j.thromres.2020.04.024 32353746 PMC7177070

[59] Baldini T Asioli GM Romoli M Carvalho Dias M Schulte EC Hauer L. Cerebral venous thrombosis and severe acute respiratory syndrome coronavirus-2 infection: A systematic review and meta-analysis. Eur J Neurol. (2021) 28:3478–90. 10.1111/ene.14727 33426733 PMC8014715

[60] Abouhashem S Eldawoody H Taha MM. Cerebral venous sinus thrombosis in patients with COVID-19 infection. Interdisciplinary Neurosurgery. (2021) 24:101091. 10.1016/j.inat.2021.101091 33520667 PMC7834014

[61] Hemasian H Ansari B. First case of Covid-19 presented with cerebral venous thrombosis: a rare and dreaded case. Rev Neurol. (2020) 176:521. 10.1016/j.neurol.2020.04.013 32414532 PMC7211601

[62] Thompson A Morgan C Smith P Jones C Ball H Coulthard EJ. Cerebral venous sinus thrombosis associated with COVID-19. Pract Neurol. (2021) 21:75–6. 10.1136/practneurol-2020-002678 33033161

[63] Hughes C Nichols T Pike M Subbe C Elghenzai S. Cerebral venous sinus thrombosis as a presentation of COVID-19. Eur J Case Rep Intern Med. (2020) 7:001691. 10.12890/2020_001691 32399457 PMC7213833

[64] Ulivi L Squitieri M Cohen H Cowley P Werring DJ. Cerebral venous thrombosis: a practical guide. Pract Neurol. (2020) 20:356–67. 10.1136/practneurol-2019-002415 32958591

[65] Xian Z Chen Y Chen L Lu Q Huang G Qin Q. A clinical research on the potential pathogenesis of somatic cancer related cerebral venous sinus thrombosis. Medicine. (2019) 98:e15134. 10.1097/MD.0000000000015134 31083150 PMC6531122

[66] Elyamany G Alzahrani AM Bukhary E. Cancer-associated thrombosis: an overview. Clinical Medicine Insights: Oncology. (2014). 8:CMO-S18991. 10.4137/CMO.S18991 25520567 PMC4259501

[67] Ostovan VR Foroughi R Rostami M Almasi-Dooghaee M Esmaili M Bidaki AA. Cerebral venous sinus thrombosis associated with COVID-19: a case series and literature review. J Neurol. (2021) 22:1–2. 10.1007/s00415-021-10450-8 33616740 PMC7897893

[68] Borhani-Haghighi A Ghannadi S Safari A Neydavoudi M Poursadeghfard M Ashjazadeh N. The study of intermediate-term survival of the patients with cerebral venous sinus thrombosis. Neurology Asia. (2020) 25:453–7. Available online at: https://www.neurology-asia.org/articles/neuroasia-2020-25(4)-453.pdf

